# Risky sexual behaviour among Russian adolescents: association with internalizing and externalizing symptoms

**DOI:** 10.1186/s13034-021-00393-3

**Published:** 2021-08-09

**Authors:** Johan Isaksson, Caroline Westermark, Roman A. Koposov, Andrew Stickley, Vladislav Ruchkin

**Affiliations:** 1 Child and Adolescent Psychiatry Unit, Department of Neuroscience, Uppsala University, 751 85 Uppsala, Sweden; 2 Department of Women’s and Children’s Health, Pediatric Neuropsychiatry Unit, Centre for Neurodevelopmental Disorders at Karolinska Institutet (KIND), Karolinska Institutet, Stockholm, Sweden; 3 Regional Centre for Child and Youth Mental Health and Child Welfare, UiT-The Arctic University of Norway, Tromsö, Norway; 4 Sechenov First Moscow State Medical University, Moscow, Russia; 5 The Stockholm Center for Health and Social Change (SCOHOST), Södertörn University, Huddinge, Sweden; 6 Department of Preventive Intervention for Psychiatric Disorders, National Institute of Mental Health, National Center of Neurology and Psychiatry, Tokyo, Japan; 7 Child Study Centre, Yale University School of Medicine, New Haven, CT USA; 8 Säter Psychiatric Clinic, Säter, Sweden

**Keywords:** Internalizing symptoms, Externalizing symptoms, Risky sexual behaviour, Adolescents

## Abstract

**Background:**

Risky sexual behaviour (RSB) is regarded as a major health problem during adolescence. Russia has one of the highest rates of teenage pregnancy, abortion and newly diagnosed HIV infections in the world, but research on RSB in Russian youth has been limited. To address this deficit, this study examined the role of several factors, including internalizing and externalizing symptoms, in RSB among Russian adolescents.

**Methods:**

Self-reported data were collected from 2573 Russian adolescents aged 13–17 years old (59.4 % girls; Mean age = 14.89) regarding RSB (unprotected sex, early pregnancy, multiple sexual partners and substance use during sexual encounters). Information was also obtained on externalizing (conduct problems and delinquent behaviour) and internalizing (depression, anxiety and posttraumatic stress) symptoms, as well as interpersonal risk and protective factors (affiliation with delinquent peers, parental involvement and teacher support). Hierarchical multiple binary logistic regression analysis was used to examine the associations between these variables and RSB.

**Results:**

Boys reported engaging in more RSB than girls. Externalizing symptoms and affiliation with delinquent peers were most strongly associated with RSB, whereas symptoms of anxiety were negatively associated with RSB. There was an interaction effect for sex and affiliation with delinquent peers on RSB with boys reporting RSB when having more delinquent peers. Neither parental involvement nor teacher support were protective against RSB.

**Conclusions:**

Early detection of and interventions for RSB and associated externalizing symptoms may be important for adolescent physical and mental wellbeing. Affiliation with delinquent peers should, especially among boys, be regarded as a risk marker for RSB.

## Background

Risky sexual behaviour (RSB) during adolescence including unprotected sex, multiple sexual partners and substance use during sexual encounters [1–4] is regarded as a major public health problem, with increased risk for unplanned pregnancy and sexually transmitted infections (STIs) [5]. Pregnancy during adolescence has been associated with more complications for female health, as well as an increased risk for preterm delivery, low birth weight and severe neonatal conditions [6], while STIs may result in other complications such as ectopic pregnancy, miscarriage and congenital infections [7]. RSB has also been linked to a number of negative social consequences including lower school grades [8] and an increased risk for sexual victimization in adolescent girls [9]. Former Soviet countries have some of the highest teenage pregnancy and abortion rates [10] and Russia has one of the highest rates of newly diagnosed HIV infections in the world, which is further complicated by the relatively low treatment coverage and shortage of treatment strategies [11], and increasing infection rate among youths [12]. However, research exploring the factors that may underlie adolescent RSB in Russia has been limited. This is of particular concern given that the prevalence of RSB varies between countries, and is often affected by the social and economic situation, such a social norms, public policies, and poverty [13].

Previous research, primarily in western societies has linked poor mental health conditions to a wide range of risk behaviours, including RSB. Mental health conditions in children and youth are often classified into two broad transdiagnostic dimensions: externalizing and internalizing symptoms [14, 15]. Externalizing symptoms are characterized by behavioural disinhibition or disruptive behaviour and include conduct problems, hyperactivity and antisocial behaviour. These symptoms are thought to be the result of such factors as under-controlled or disinhibited behaviour and may serve as major risk factors for substance use, later adult crime and violence [16, 17]. Internalizing symptoms refer to negative mood states and inhibition, and in contrast to externalizing problems, are linked to over-controlled behaviour [18] and include symptoms of depression, anxiety and posttraumatic stress disorder (PTSD). Importantly, even though externalizing and internalizing symptoms relate to different symptom clusters, co-morbidity is common [14, 15], and there is a need to adjust for both clusters in order to evaluate any unique impact of these symptoms on risk behaviours.

Previous studies have mostly stressed the importance of externalizing symptoms for RSB during adolescence, with reports of a broader range of externalizing symptoms being associated with more RSBs, including the number of sexual partners, frequency of using protection and frequency of drug and alcohol use during sex [19]. In addition, delinquency in early adolescence has been associated with having multiple sexual partners, an STI diagnosis [20] and with early sexual initiation [21], which itself is associated with future RSB [22]. Children and adolescents with inattention and hyperactive symptoms initiate intercourse earlier, have more sexual partners, and more partner pregnancies [3, 23]. Lower self-control and conduct problems during childhood have been linked to an increased risk for multiple sexual partners and early parenting [24]; while sensation seeking and affiliation with deviant peers at age 12 predict an earlier initiation of intercourse, sex without protection and partner pregnancies [25].

The literature on the association between internalizing symptoms and RSB has produced mixed findings, with anxiety symptoms being associated with both a decreased [21] and increased risk of early sexual initiation during adolescence [26]. Studies on depression, on the other hand, have demonstrated a more consistent pattern, indicating that depressive symptoms are associated with early sexual initiation [8, 26, 27] as well as a broader range of RSBs [28]. Surprisingly, there is a shortage of studies investigating the association between PTSD and RSB among adolescents, however, among young adult populations, more PTSD symptoms have been shown to predict the number of sexual partners and risky/impulsive sexual behaviours [29]. In addition, interpersonal factors may exert both risk and protective effects on RSB in adolescence. For example, affiliating with delinquent peers has been shown to be a risk factor [2, 20, 25] whereas family and teacher involvement/support has been linked to a decreased risk for RSB [2].

RSB is more common among adolescent males than females [21, 30]. This sex difference may be partly explained by gender norms, where females are expected to abstain from sex to a greater degree than males [31], but might also be related to sex differences in the prevalence of mental health conditions, with externalizing symptoms being more commonly observed amongst boys, whereas internalizing symptoms [32] and disproportionate health consequences related to STIs, including pelvic inflammatory disease, infertility, and unintended pregnancy [5] are more prevalent among girls. Despite this research on sex differences in RSB and mental health conditions, there has been a shortage of studies examining possible sex-specific associations between externalizing and internalizing symptoms and RSB, i.e. on whether externalizing and internalizing symptoms contribute differently to RSB in boys and girls. Depressive symptoms have been shown to predict having more sexual partners among boys, but less frequent use of protection among girls in a longitudinal study conducted among U.S. adolescents [33], while internalizing problems at ages eight and ten have been associated with early sexual intercourse for boys but not girls [34]. In contrast, PTSD symptoms have been associated with unprotected intercourse among adolescent girls, but not boys [35]. With regard to externalizing symptoms, in a meta-analytic review the association between impulsivity and adolescent RSB was stronger in samples with more females [36]. In contrast, Thijs et al. [37] found that the association between conduct problems and problematic behaviour in adolescence, including having sexual intercourse, was stronger for boys than for girls, while Skinner et al. [38] showed that childhood externalising behaviour problems predicted having had more multiple sexual partners by age 17 for boys but not girls. However, in the same study, only girls with childhood externalising behaviour problems were more likely to have had unwanted sex [38]. In short, previous findings on the association between mental health conditions and RSB are mixed and more studies are needed in order to determine any sex-specific effects in the associations.

Given the paucity of studies investigating the sex-specific pattern in the association between mental health symptoms and RSB among adolescents in non-western cultures, the current study used data from Russia, to examine the role of individual risk factors such as internalizing (depression, anxiety and posttraumatic stress) and externalizing (conduct problems and delinquent behaviour) symptoms and interpersonal factors such as delinquent peers, parental involvement and teacher support in the occurrence of RSB. We hypothesized that (i) externalizing symptoms will be more strongly associated with RSB than internalizing symptoms and (ii) boys will report more RSBs than girls. We have refrained from any hypotheses regarding potential sex-specific patterns in the association between externalizing/internalizing symptoms and RSB.

## Methods

### Participants and procedures

Data comes from the Social and Health Assessment (SAHA), a research project that investigated factors associated with adolescent health and wellbeing [39, 40]. The study was undertaken in Arkhangelsk, a large city in the North-western part of European Russia, that had a population of approximately 350,000 inhabitants at the time of the study (2003), and a socioeconomic status (SES) in the low to average range compared to the rest of Russia. A two-stage selection procedure was used to obtain a representative sample of adolescents in the desired age range. From the 71 eligible public schools (schools with special education programmes were excluded), 14 were randomly selected, resulting in 210 classes. In total, responses were collected from 2847 students. Of these, 42 students were excluded for being outside the required age range (13–17 years) and 232 students were excluded as a result of missing data. Data from a total of 2573 adolescents aged 13–17 years old (59.4 % girls; M = 14.89, SD = 1.12) thus comprised the analytic sample. Those excluded due to missing data on any of the included variables had higher ratings on affiliation with delinquent peers (*t* = 2.22; *p* = .026; df = 2801), and lower SES (*t* = 2.67; *p* = .007; df = 2762) and teacher support scores (*t* = 2.12; *p* = .034; df = 2802). Furthermore, there was a higher number of boys among those excluded (*χ²* = 23.83; *p* < .001; df = 1).

The survey questionnaire was translated into Russian following established guidelines, including appropriate use of independent back translations [41]. All questionnaires were pre-tested in different samples of youths. The survey was completed by the students in their classrooms during a normal school day. Before the survey administration, written informed consent was obtained from all participants, with both parents (for the child) and the students themselves having the right to refuse to participate in the study. The study was approved by appropriate institutional review committees.

### Measures

#### Risky sexual behaviour (dependent variable)

Four questions were used as indicators of RSB [(i)–(iv)]: (i) “How many people have you had sexual intercourse with?”, with two or more sexual partners (as suggested by e.g., Fleming et al. [2]) being categorized as RSB (1/0), (ii) “The last time you had sexual intercourse, had you been drinking alcohol or using drugs?” (yes [1]/no [0]), and (iii) “The last time you had sex did you or your partner use a condom?” (no [1]/yes [0]). Although pregnancy in adolescence is not a RSB per se, but rather a consequence of it, we included an additional question: (iv) “How many times have you been pregnant or got someone pregnant?” with one or more pregnancies regarded as a proxy for RSB. These four questions have also been used previously to define RSB [3]. The total sum score could range between 0 and 4, with higher scores indicating more RSB. Cronbach’s alpha for the scale was 0.64. For the main analysis the score was recoded into a dichotomous variable, where any RSB was coded as “1” and no RSB as “0”.

#### Fixed risk factors

Fixed markers included *sex* and *age*. In addition, SES was assessed on current parental employment for each parent separately: unemployed (0), part-time (1), full-time (2), with a possible score between 0 and 4 for each family.

### Individual risk factors

*Externalizing symptoms* were measured using a scale designed to assess conduct problems and delinquent behaviour of different severity [40]. The students answered on a five-point scale how many times they had been involved in specific behaviours in the past year where 0 = zero times, 1 = once, 2 = twice, 3 = three or four times, 4 = five or more times. The scale consisted of 19 items ranging from moderately mild conduct problems (e.g. staying out all night without permission, lying to a teacher or parent, damaging property, shoplifting and skipping school), to non-violent antisocial behaviours (e.g. pickpocketing, stealing a motorcycle or a car and being high at school as a result of drinking alcohol or smoking marijuana), through to relatively serious aggressive and antisocial behaviour (e.g. starting a fistfight, hurting somebody badly in a fight, participating in a gang fight, carrying a blade or knife to school and suffering legal consequences as a result of antisocial behaviour). The total scale score could range from 0 to 76 with higher scores indicating more externalizing behaviour symptoms. The scale had a good level of internal consistency (Cronbach’s alpha was 0.87).

*Internalizing symptoms* were assessed with three self-rated scales, targeting three different types of conditions within the spectrum. *Depressive symptoms* were measured using a modified version of the Centre for Epidemiologic Studies-Depression Scale (CES-D) [42]. Both the CES-D [43] and its modified and shortened versions [44] have been found to have excellent psychometric properties when used in adolescent populations. The 10-item scale consists of questions on symptoms associated with depression (e.g. feeling lonely, feeling disliked and having problems sleeping). Students answered using a three-point scale: Not true (scored 0), somewhat true (1), certainly true (2) in relation to possible symptoms during the past month. Scores were summed and could range between 0 and 20, with higher scores indicating an increased number of depressive symptoms. Cronbach’s alpha for the scale was 0.82.

*Anxiety symptoms* were assessed using a 12-item scale [40] which measures the cognitive-affective and behavioural types of anxiety. Specifically, this related to worrisome and preoccupying thoughts or unpleasant feelings about the student him/herself or about external stimuli (e.g. feeling nervous when being called on in class, worrying about the future or worrying whether other people like her/him). The students reported these symptoms using a three-point scale: Not true (scored 0), somewhat true (1), certainly true (2). The total score could range between 0 and 24, with higher ratings indicating a higher number of anxiety symptoms. Cronbach’s alpha for the scale was 0.86.

*Posttraumatic stress symptoms* were assessed using the Child Post-Traumatic Stress-Reaction Index (CPTS-RI); a 20-item scale used with children and adolescents to evaluate reactions after exposure to a broad range of traumatic events (e.g. questions about nightmares, sleep, behavioural manifestations). Each item was rated on a five-point scale: Never (scored 0), seldomly (1), sometimes (2), much of the time (3), almost always (4). The total summed score could range between 0 and 80, with higher scores indicating more severe PTSD symptoms [45]. Previous research has indicated that the scale correlates highly with DSM-based diagnoses of posttraumatic stress syndrome [45, 46]. The scale had a Cronbach’s alpha value of 0.84.

### Interpersonal risk and protective factors

*Affiliation with delinquent peers* was assessed using a nine-item scale [40], that asked students about how many of their friends were involved in risk taking behaviours, such as dropping out of school and smoking cigarettes. Each item was rated on a four-point scale ranging from “none” (scored 1) to “most or all” (scored 4). The total summed score could range from 9 to 36 with higher scores indicating more friends engaging in antisocial behaviour. Cronbach’s alpha for the scale was 0.87.

*Parental involvement* was measured with six items [40] enquiring about students’ perceptions of parenting practices (e.g. “asks about her/his life”, “gives good advice”). The students reported on a four-point scale, ranging from “never” (scored 1) to “often” (scored 4). The total summed score could range from 6 to 24 with higher scores indicating more parental involvement in their children’s lives. Cronbach’s alpha for the scale was 0.81.

*Teacher support* was assessed with the school environment and academic motivation scale [40], consisting of eight items (e.g. “teachers are willing to help students”; “most of my teachers notice when I am doing a good job and let me know about it”). Each item was rated on a four-point scale in terms of how true the statement was for them (ranging from 1 = “definitely not true” to 4 = “definitely true”). The total summed score could range from 8 to 32 with higher scores indicating more teacher support. Cronbach’s alpha for the scale was 0.64.

### Statistical analyses

Data were analysed using the Statistical Package for the Social Sciences (SPSS-26). Chi-square tests were used for univariate comparisons of different forms of RSB between girls and boys, and independent sample t-tests were used for comparing ratings on the study variables between those with and without RSB. RSB as a dichotomous outcome variable was regressed on fixed markers, individual- and interpersonal-factors using a hierarchical multiple binary logistic regression model in four steps where only variables that were significantly associated with any RSB in the logistic regression were included in the model; Step 1 adjusted for fixed factors; Step 2 additionally adjusted for internalizing symptoms; Step 3 further adjusted for externalizing symptoms; in Step 4 interpersonal risk and protective factors, i.e., affiliation with delinquent peers, teacher and parental involvement, were also adjusted for. Potential sex-specific effects of all included interpersonal risk and protective factors for RSB were investigated as a post-hoc analysis using interaction terms in the adjusted model. The results are presented as odds ratios (OR) with 95 % confidence intervals (CI). Two-tailed tests with p-values < 0.05 were considered as being statistically significant.

## Results

### Association between RSB and the other study variables

In total, N = 538 (20.9 %) students had engaged in at least one RSB, with a higher frequency amongst boys compared to girls. Descriptive data on sex differences in RSB are presented in Table 1. Boys more frequently reported using alcohol or other drugs during last intercourse and having multiple (≥ 2) sexual partners. As shown in Table 2, those reporting any RSB were older, had more externalizing, depressive and posttraumatic stress symptoms, and more delinquent peers. In contrast, those with RSB reported fewer symptoms of anxiety, less parental involvement and teacher support.

**Table 1:** Sex differences in risky sexual behaviour among Russian adolescents

	Boys n (%)	Girls n (%)	Total n (%)	Statistics *χ2* (p-value)
One or more RSB	298 (28.5)	240 (15.7)	538 (20.9)	61.58***
Having had sex with two or more persons	213 (20.9)	97 (6.4)	310 (12.3)	118.00***
Using alcohol or drugs during last intercourse	109 (10.6)	53 (3.5)	159 (6.4)	52.14***
Sex without condom at last intercourse	119 (11.6)	166 (10.9)	285 (11.2)	0.25
Having been pregnant or partner pregnancies	64 (6.4)	91 (6.1)	155 (6.2)	0.08

Reference values are no RSB (i.e., sex ≤ 1 person, not having sex or not using alcohol, drugs or condom during last intercourse, and not reporting pregnancies)

*RSB* risky sexual behaviour

*** *p* < .001

**Table 2:** Mean and standard deviations (SD), and differences on the study variables by risky sexual behaviour among Russian adolescents

	RSB (N = 538) Mean (SD)	No RSB (N = 2035) Mean (SD)	Statistics
Age	15.33 (1.06)	14.77 (1.09)	*t* = 10.60***
Socioeconomic status (range 0–4)	3.11 (1.10)	3.09 (1.10)	*t* = 0.28
Externalizing symptoms (range 0–76)	14.08 (14.12)	5.68 (6.69)	*t* = 19.74***
Depressive symptoms (range 0–20)	6.43 (4.41)	5.84 (4.13)	*t* = 2.91**
Anxiety symptoms (range 0–24)	11.45 (5.16)	12.81 (5.35)	*t* = 5.29***
Posttraumatic stress symptoms (range 0–80)	21.09 (11.99)	19.53 (11.17)	*t* = 2.83***
Affiliation with delinquent peers (range 9–36)	21.59 (6.62)	17.23 (5.55)	*t* = 15.50***
Parental involvement (range 6–24)	16.64 (3.62)	17.26 (3.55)	*t* = 3.61***
Teacher support (range 8–32)	20.40 (4.29)	21.25 (4.07)	*t* = 4.28***

*RSB* risky sexual behaviour

**p < .01; ***p < .001

### Adjusted model (logistic regression)

In a hierarchical binary logistic regression analysis, where non-significant associations were subsequently removed (i.e., SES, parental involvement and teacher support), the final model explained 25 % of the variance in RSB. As shown in Table 3, in the final model the presence of RSB was predicted by male sex, increasing age, externalizing symptoms, affiliation with delinquent peers, whereas anxiety was associated with lower odds for RSB. In addition, an interaction effect was found for sex and affiliation with delinquent peers on RSB (OR = 1.06, 95 % CI = 1.02, 1.10, *p* = .003), with boys (OR = 1.09, 95 % CI = 1.06, 1.13, *p* < .001) but not girls (OR = 1.02, 95 % CI = 0.99, 1.05, *p* = .15) significantly more likely to report RSB when having more delinquent peers.

**Table 3:** Results of a hierarchical binary logistic regression analysis predicting any risky sexual behaviour as a dichotomous outcome in adolescents

	Step 1	Step 2	Step 3	Step 4 (final model)
	OR (95 % CI)	OR (95 % CI)	OR (95 % CI)	OR (95 % CI)
Sex (Ref = Females)	2.47 (2.02–3.01)***	2.55 (2.07–3.14)***	1.78 (1.43–2.23)***	1.73 (1.39–2.17)***
Age	1.66 (1.52–1.82)***	1.62 (1.48–1.78)***	1.57 (1.42–1.73)***	1.51 (1.37–1.67)***
Depressive symptoms		1.04 (1.01–1.07)*	1.01 (0.97–1.04)	1.01 (0.98–1.04)
Anxiety		0.94 (0.93–0.96)***	0.97 (0.95–0.99)**	0.97 (0.95–0.99)**
Posttraumatic stress		1.02 (1.01–1.03)***	1.01 (1.00-1.02)	1.01 (1.00-1.02)
Externalizing symptoms			1.08 (1.07–1.09)***	1.06 (1.05–1.08)***
Delinquent peers				1.06 (1.03–1.08)***
Model R^2^	0.11	0.14	0.23	0.25

The Step 1 analysis included significant fixed markers of risk; In the Step 2 analysis significant internalizing (depressive, anxiety and posttraumatic stress) symptoms were added to the variables included in Step 1; The Step 3 analysis added significant externalizing symptoms (conduct problems and delinquent behaviour) to the variables included in Step 2; The fully adjusted Step 4 analysis added affiliation with delinquent peers (i.e., how many of their friends were involved in risk taking behaviours) to the variables included in Step 3

*CI* confidence interval, *OR* odds ratio, *R*^2^ Nagelkerke R square

*p < .05, **p < .01, ***p < .001

## Discussion

In this study of Russian adolescents, RSB was more commonly reported by boys compared to girls, and it was predicted by externalizing symptoms and affiliation with delinquent peers, whereas anxiety symptoms were negatively associated with RSB. In addition, for boys there was an interaction effect for sex and affiliation with delinquent peers, with boys having higher odds for RSB when associating with more delinquent peers. Neither parental involvement nor teacher support were protective factors in the adjusted model.

In line with our first hypothesis, externalizing symptoms were most strongly associated with RSB. This result concurs with previous studies that have investigated the association between RSB and externalizing symptoms [3, 19–21, 23–25]. According to the Problem-Behaviour Theory the association between externalizing symptoms and RSB relates to multiple psychosocial risks including personality characteristics (e.g. high tolerance of deviance), social environmental factors (e.g. peer pressure) and other behaviours (e.g. low school achievement) [47]. Others have focused on the potential importance of individual factors such as impulsivity when discussing this association [48]. Both externalizing symptoms and RSB may also be regarded as a natural part of adolescence rather than as “problem behaviours”, through which adolescents may strengthen their identity and find self-worth [49]. However, even though a certain level of risk-taking behaviour may serve as a natural developmental factor for adolescents, it does not diminish the possible negative consequences of RSB such as STIs and HIV infection [5]. This is of special concern in Russia, with one of the highest rates of newly diagnosed HIV infections in the world [11].

Even though internalizing symptoms were not as strongly associated with RSB as externalizing symptoms, a positive association was observed between depressive symptoms and RSB, which corroborates the findings of previous studies [8, 26–28]. However, the association became non-significant in the adjusted model, underscoring the importance of adjusting for co-morbidity given the high degree of overlap between externalizing and internalizing symptoms [14, 15]. Interestingly, anxiety symptoms were negatively associated with RSB in our study sample, with the association remaining significant even in the fully adjusted model. Anxiety has been linked to a reduced likelihood of initiating sexual activity among early adolescents in the U.S [21], whereas in a sample of European adolescents anxiety was associated with a greater risk of early sexual initiation [26]. Hypothetically, these conflicting results regarding anxiety and RSB may be potentially explained by the different functions of intimacy or intercourse, which in certain circumstances may serve as a means to release anxiety whereas in other circumstances, increased anxiety could potentially reduce engagement in risk-taking behaviour as some types of anxiety, e.g. social anxiety, may result in avoidance of social and intimate situations due to the fear of negative evaluation. In order to better understand the link between anxiety and RSB, future studies would benefit from adopting a longitudinal approach.

As hypothesised, boys reported more RSB in comparison to girls, which is also in line with earlier studies [21, 30]. In particular, boys were significantly more likely to have had more than one sexual partner than girls, and have used alcohol or drugs during last intercourse. This might be explained, at least in part, by different attitudes towards sexuality between the genders, where societal norms are manifest in the expectation that females to a higher degree than males will abstain from sex [31]. In addition, as it is considered more normative for boys to be sexually active than girls it is possible that they may also be more willing to report RSB compared to girls. Hypothetically, these sex differences in RSB might also be related to the higher frequency of externalizing symptoms amongst boys [32], however, male sex predicted RSB even when adjusting for externalizing symptoms.

Affiliation with delinquent peers was associated with RSB in our study, which corroborated previous research [2, 20, 25]. Factors such as peer pressure may possibly result in teenagers adjusting to the behavioural norms typical for their environment, including norms about sex, drugs and delinquency, which in turn may lead to risky behaviour becoming normalized [50]. We found an interaction effect for sex and affiliation with delinquent peers, where boys with more delinquent peers reported more RSB. A similar finding was reported by McCoy et al. [50] in a review study where gender differences in adolescent susceptibility to deviant peer pressure were reported in almost half of the included studies, with boys being more susceptible to deviant peer pressure for risk-taking behaviours than girls. The authors discussed this finding as a possible result of gender socialization where masculinity is linked with toughness and autonomy and boys’ social environments are more tolerant and include less adult supervision, enabling affiliation with delinquent peers [50].

Our study has several strengths, including the use of a large sample of community-based adolescents from Russia and being able to assess a variety of internalizing and externalizing variables. Nonetheless, there are some limitations that need to be mentioned. There are several potential difficulties when assessing sexual behaviour and psychosocial factors amongst adolescents. To solely rely on self-reported screening methods may result in different forms of bias such as recall bias that relates to participants’ ability to accurately recall past events, and social desirability bias which is connected to participants’ ability to report truthfully on sensitive topics [51]. A disbelief in the confidentiality of the survey could also have affected the answers. Even though there is some evidence that self-reported information on potentially sensitive topics such as teenage drinking habits is generally valid [52], for future studies, the inclusion of parental and teacher ratings may help facilitate a more valid and comprehensive understanding. In addition, although conduct problems and delinquent behaviour were used as a proxy for externalizing symptoms, including symptoms of inattention and hyperactivity may have provided additional information. As this study used cross-sectional data it was not possible to determine the temporal nature of the observed associations or establish causality, and hypothetically RSB may by itself increase the risk for externalizing problems and affiliation with delinquent peers. Future studies would benefit from employing longitudinal cross-cultural designs, in order to assess if mental health conditions are predictors for RSB, while also looking at the sex-specific patterns in the relation. Lastly, we lacked information on other factors that might have affected the association between internalizing and externalizing behaviours and RSB such as social attitudes and norms towards RSB.

In conclusion, higher levels of externalizing symptoms were found to predict RSB. In contrast, higher levels of anxiety were associated with reduced odds of engaging in RSB. A sex-specific pattern regarding the effect of delinquent peer affiliation on RSB was observed where boys who associated with more delinquent peers were significantly more likely to engage in RSB. Considering the negative consequences that RSB may have on adolescent sexual and reproductive health it is of the utmost importance that RSB and externalizing symptoms be detected and assessed as early as possible amongst adolescents. This is especially important in societies such as Russia with high rates of teenage pregnancy, abortion and newly diagnosed HIV infections, and with low treatment coverage [10, 11].

## Data Availability

Data are not in the public domain but requests can be sent to the corresponding author for consideration.
